# Reviews of Books

**Published:** 1924-04

**Authors:** 


### REVIEWS OF BOOKS.

### A LADY DOCTOR IN CONSTANTINOPLE.

**From a Balcony on the Bosphorus.** By A. Louise McIlroy. Illustrated. ("Country Life." 5s. net.)

It need hardly be explained that Dr. McIlroy is Professor of Obstetrics and Gynæcology at the Royal Free Hospital School of Medicine for Women. Her charming little book has, however, not much to say about hospital matters in Stamboul; the rather is it a vivacious account of scenes and life in that city during the Armistice period of 1919–20. It is difficult to write anything new about Constantinople, the strange city in which Europe and Asia meet, but Dr. McIlroy went there with observing eyes, and in many a simple yet vivid touch she paints the mingled beauty and squalor which create so many violent contrasts. She tells of her wanderings in bazaars, her visits to mosques, her watchings of the Dancing Dervishes—"graceful, silent, and devout performers," so startlingly different from the sordid proceedings of the Howling Dervishes, who "have the appearance of ruffians brought in as supers for a show which is faked solely to excite the interest of the foreigner." Since these gentry charge "a considerable fee" for the pleasure of seeing them cut their faces with knives and hearing them howl like the possessed, the suspicion that the whole business is a show seems to be fairly well founded.

Dr. McIlroy writes with charity and tolerance about the people and their ways, their faith, their politics, and their morals, which, she realises, must be judged from the Asiatic and not from the European standpoint. "To live in the Near East is to learn, and one soon recognises the difficulties that arise from the complication of religious with political affairs"; and her conclusion is that "the history of the Greek Orthodox Church is one of tyranny on the part of its Patriarch as great as that of any Ottoman Sultan"—a little bombshell which may be expected to produce a retaliatory bombardment from certain quarters. In "the daughters of Islam," who wear Paris frocks and no longer recline on cushions, Dr. McIlroy is naturally especially interested, and she tells pleasantly of their gradual emancipation. They appear even, little by little, to be acquiring the right to have souls. If they are deemed worthy they may reach immortality. In the meantime they attend classes on the Koran and sit in a gallery in the mosque—far back "so as not to be a disturbing element to the male worshipper," who otherwise might be unable to withstand the "large brown eyes" which so greatly charmed Dr. McIlroy. As we have said, hospital matters are not prominent in this beautifully printed little book, but obviously they could not be left untouched. Thus we read:—

> On the hill above Scutari is the hospital which was built to the memory of Florence Nightingale. Near it is the Turkish Military School of Medicine and its hospital across the road. This medical school was built by Abdul Hamid at a distance from the other hospital centres in Stamboul, as it was deemed more expedient to place the medical students (who are always revolutionary in their tendencies) as far away as possible from the precincts of the Yildiz and Dolma Bagtche palaces. The college has an imposing appearance, with its grand entrance-hall and staircase ornamented with arabesques. Its long corridors give it an air of spaciousness and dignity. A favourable impression is produced by the courteous reception on the part of the white-haired Principal and his staff, who over coffee and cigarettes bid the visitor welcome. The equipment of the laboratories is quite up to date, and compares favourably with many of our Western schools. The hospital wards are clean, with large open windows, and here we find white-capped little nurses, modest and retiring, who give a modern touch to an Eastern land. The medical staff are most anxious to adopt the latest scientific methods, and they have an Englishwoman as matron at the head of the hospital for the training of the nurse probationers.

What hospitals mean in a city like Constantinople, especially in times when the aftermath of war is being gathered, is illustrated in another interesting passage:—

> Although each army had its own military hospitals, accommodation was not reserved entirely for soldiers, and civilians were permitted to take advantage of the services of their medical staffs. . . . The officers' section of the British Military Hospital, taken over from the Germans, was found to be extremely up-to-date and handsomely equipped on the most modern lines. It had been in existence for a number of years before the war, and was one of the most important and indeed commendable pieces of propaganda work carried out by the Germans in Turkey. The English-speaking community of Constantinople after the war came to the hospital for advice and treatment, and patients from far-off Turkish villages had the advantage of its specialist staff. Accident cases picked up from the streets, little children starving and dying by the road-side, and refugees from Russia worn out in body and despairing of soul—all were given a helping hand.

It is a book marked by acute observation, is thoroughly well written, and full of the graphic little touches which bespeak the cultured woman easily assimilating the unfamiliar.

### MAN, THE MONGREL.

**Heredity and Eugenics.** By R. Ruggles Gates, Ph.D. (Constable. 21s. net.)

So much are the theories of heredity and human evolution part and parcel of the current thought of the day that to the present generation there seems an air of unreality about the fierce controversy which raged round the cradle of the *Origin of Species*. Yet, though our knowledge of the facts of heredity and its cognate subjects has increased ten thousand fold, these facts seem only to add to the intricacy and difficulty of their interpretation. Weismann's exposition of the non-inheritance of acquired characters, and the re-discovery of Mendel's experimental investigations gave the first heavy blows to the structure which appeared to rest on so sure a foundation in natural selection. To-day, when we consider the problem of human inheritance, we find ourselves with the space already cleared for the new building and the stones cut and squared ready to our hand, but with no very clear idea of the structure we shall ultimately raise. The old and often entirely misleading antithesis of Nature *versus* nurture, heredity *versus* environment, is but an indication of the complexity of the problem. In the course of his social evolution the character of man's environment has changed so greatly that the race is no longer to the swift nor the battle to the strong, and the struggle for existence has shifted its arena and become preponderatingly ethical rather than physical. Hence any discussion of human heredity must take the social aspect of the question into serious consideration.

As a professor of botany, Dr. Gates has served an extended apprenticeship in those particular aspects of heredity which are best revealed in plant life, and though his equipment in the field of human development is necessarily less complete, he has collected a large mass of facts which have a bearing upon the problem. By the increasing complexity of the environment in which he has lived, and by his triumph over the forces of Nature, man has ceased to conform to the strict law of the survival of the fittest, and in consequence of his long-continued miscegenation he is, speaking biologically, too mongrel to allow the application of the same strict canons of observation which can be applied to other animals and to plants. Hence the patient accumulation and sifting of many more observations is required before the tangled skein of his inheritance will be unravelled.

Admirable as is Dr. Gates's volume as a whole, he has been led into certain minor errors, especially in reference to those individuals who exhibit that lack of mental capacity which we label "feeble-mindedness." There are grounds for believing that this is not a unitary condition, but that we group several distinct states under this term. Hence, without a clear definition and understanding of the expression, such a phrase as "the mental level of the average Palæolithic man can hardly have been higher than that of our modern feeble-mindedness" can have no real meaning. A similar confusion of thought is seen in the quotation of Goddard's highly misleading statement that "the average steerage passenger immigrant into the United States is of low intelligence, perhaps even feeble-minded," or that the mental age of twelve years is to be regarded as the line between feeble-minded and normal. Further, it is quite misleading to say that the mentality of a Mongol child is nearly always that of a four-year old child, and that this type of abnormality is a form of hypohyroidism. There is probably a good deal of truth in the theory that Crétinism and Mongolism and some of the forms of idiocy are lethal variations. The book is a useful contribution to a subject of absorbing interest, and will be read with profit by all who believe in the progress and betterment of mankind.

### HIGH FREQUENCY AND OTHER MATTERS.

**High Frequency Practice for Practitioners and Students.** By Burton Baker Grover, M.D. (Chapman and Hall. 21s.)

The American author is too modest in his choice of title; this book contains much useful information and philosophy on general medicine and the preservation of health as well as on high frequency. A successful attempt has been made to give a physiological reason for the use of high frequency currents which the author seldom recommends on empirical, rule-of-thumb principles. The English reader, unfamiliar with American words and spelling, will occasionally be brought up with a jerk when he encounters such a sentence as: "Many cases of acute bronchitis may be jugulated by early use of diathermy." The sensation of being "jugulated" may be intriguing and euphoric, but most English readers would like to know more precisely what to expect of the early use of diathermy in acute bronchitis. There are, of course, many technical words familiar only to the electrician, and the author has thoughtfully supplied a glossary at the end of the book. Human arteries, we are told, are not unlike in efficiency the garden hose sold by the shopkeeper; one grade will last one season, another two seasons, while the best grade will last for years. Commonsense and practical illustrations such as this add appreciably to the appeal which the book makes to the average reader who has not yet learnt to think of the human body in terms of electricity.

A whole chapter is devoted to the blood pressure, and another to pain. On the causes of headache the author is most statistically explicit. He calculates that if a thousand consecutive cases of headache were to be studied, we would find 350 to be due to psychoneuroses, about 200 to Bright's disease, about 200 to high blood pressure, eyestrain and infectious diseases, 100 to meningitis, sinusitis, neuralgia and migraine, about 50 to brain tumour and syphilis, and the remaining 100 to unknown causes. One of the causes of headache in women and children is what the author calls "pituitary hunger"—a condition he defines as carbohydrate dypsomania or a craving for sweets. "A candy spree" is often followed by pituitary headache; the spree terminates, not unlike alcoholic intoxication, in nausea and vomiting. These and many other interesting passages occur in the latter part of the book, and the reader, if he is conscientious, will first qualify himself for this lighter literature by a study of the first part, which is full of technical details about electricity in general and high frequency in particular. The numerous illustrations should prove of great value in facilitating the assimilation of all this technical knowledge.

### FROM THE INSIDE.

**Scenes from Hospital Life.** Edited by Fanny Gilpin. (Drane. 3s. 6d. net.)

The proceedings of hospital life, seen from the nurse's point of view, are always of interest to a wide circle of people who, either because they have themselves been or because they contemplate becoming nurses, welcome any opportunity of getting a peep behind the scenes. No doubt, therefore, many readers besides nurses will find pleasure and interest in the lively pages of these "Letters from a Probationer Nurse." The incidents described by the author are full of life, and though a great deal is necessarily rather trivial—just the natural expression of feelings that one would expect to find in the letters of a home-bred girl writing freely to her own family circle—other passages in the book show "Prudence Herbert," the probationer, to be a shrewd observer and a keen judge of character, and her pen-pictures of some of the persons around her are both amusing and true to life.

She shows a very tender heart towards her nurse-friend, "Molly," and to the patients, but seems to have been unfortunate in her matron, who apparently is all a matron should not be. "She doesn't care a jot what happens to the nurses—their well-being is the last thing that ever enters her head," is the censure passed upon her by this severe young critic. Some of the sisters also are described with a pen dipped in gall. One of them is reported as skimming the daily supply of milk intended for ward use, and reserving the cream for herself and her pet cat, while the patients are fed with the remainder! Another sister "belongs to a religious order and goes to church every morning and evening," but spends the rest of her time in "making alterations and finding fault." A third is "cruel," and the children cry when she approaches their cots to do a dressing. Not all, however, are thus denounced. "Sister D.," writes Prudence, "is kindness itself to nurses and patients alike—an angel, and is loved by everybody." The night sister is also popular.

For the honorary medical staff there is nothing but praise. One doctor is "rather a character, but so nice. We all like him." Another is a "dear old thing, though rather pompous." A patron of the hospital, one "Mr. Manvers," is "exceedingly kind," and the book contains many references to him and to his nephew, "Dick Trevor," who is admitted to the hospital after a bicycle accident. Later he becomes the hero of the piece. In spite of all her hardships, Prudence contrives to complete her three years' training successfully, and her last letters home announce her engagement to "Dick." Whether matrons and sisters will enjoy this book as much as nurses are sure to do is a debatable point.

### THE WHOLE STUDY OF MAN.

**The Elements of Vital Statistics in their bearing on Social and Public Health Problems.** By Sir Arthur Newsholme, K.C.B., M.D. (Geo. Allen and Unwin. 24s. net.)

In the broadest sense, says Sir Arthur Newsholme, the subject of vital statistics includes the whole study of man, and although in this volume the subjects discussed are limited chiefly to those of interest to public health and social workers, the range is indeed wide and the handling of the subjects masterly and complete. The writer brings to his work not only a capacity for extensive research, and patience in the compilation of a large amount of statistical material, but also a long and wide administrative experience.

Sir Arthur Newsholme's book was first published in 1889, and has now been entirely re-written. It must be gratifying to him that urgent requests for the new edition had come from America, and he very appropriately utilises American as well as English figures and tables throughout his book. The volume indeed contains a wealth of carefully arranged information on every branch of the subject of vital statistics which can be of interest to Medical Officers of Health and all other students of public health and social welfare. For instance, in regard to infant mortality, we find an examination of various social causes of excessive mortality, such as the influence of the birth-rate, the effect of race, of density of population and overcrowding, of poverty, housing, alcoholism, and of social status, as evidenced by the occupation of the parents. In regard to statistics of general sickness, as distinct from infectious diseases, the author has not been able to glean much from the working of the National Health Insurance scheme in this country, and he does not write very hopefully of the medical records kept by panel practitioners. The records now being kept on the lines recommended by a mainly medical committee ought, however, to produce a valuable quantity of material for the use of investigators in this branch of the subject.

Not the least valuable feature of the book is the insistence everywhere upon the need, on the part of those who seek to draw inferences from statistics, for careful scrutiny of the ground covered and, in effect, for the exercise of commonsense. Illustrations—some of them very amusing—are given here and there of errors due to ratios between two variables, the comparison of incomparable items, and of drawing inferences from insufficient data. In his handling of this part of the matter, Sir Arthur Newsholme has, we suspect, the advantage of his administrative experience on which to draw. In its new form this is a thoroughly useful book.

### SMOKE ABATEMENT.

**The Smoke Inspector's Handbook; or, Economic Smoke Abatement.** By H. G. Clinch, M.R.San.I., M.I.H. (H. K. Lewis and Co., Ltd., 28 Gower Place, W.C. 1. 7s. 6d. net.)

There is an unquestioned revival of public interest in the black smoke problem and an increasing determination on the part of many public-spirited people that sunlight and cleanliness shall have free play in our towns. Here is a book on the subject full of information; the properties of fuel and the nature of smoke, the technique of draught and of the work of boilers and mechanical stokers, the law relating to emission of smoke are all set forth in clear language. The book is well printed and contains sixty illustrations. It should serve as a valuable work of reference for smoke inspectors and for medical officers of health, sanitary inspectors and public health officials generally.

It should also serve to stimulate the interest of factory owners, not only in the question of efficient appliances, but in the matter of getting the very best results out of the stokers. In an interesting preface contributed by Dr. Danks, the Medical Officer of Health for Halifax, attention is drawn to the failure of employers to recognise that it is worth while to treat boiler-firing as skilled work and to reward it accordingly. It is unquestionable that the stoker plays an important part in relation to the public health, and this book will help employers who are in earnest to get this well rubbed in, and at the same time obtain better results from their business.

---

### MISCELLANEOUS NOTICES.

#### Getting Rid of "Nerves."

Mending Your Nerves. By Flora Klickmann. (Religious Tract Society. 3s. 6d. net.)—As editor of the *Girl's Own Paper* Miss Klickmann receives every day "several letters from nerve-racked girls or women who ask what they can do to get the better of their trouble." In this little book she suggests various means of curing "nerves"; and though we should not care to adopt some of them, they are in the main full of commonsense and easy of accomplishment by women of varying circumstances and incomes. Mr. Carless,

Consulting Surgeon to King's College Hospital, contributes a preface saying that he is "impressed both by the clear appreciation of the problems raised and by the wisdom of the advice given."

### X-Ray Work.

X-Rays, Their Origin, Dosage and Practical Application. By W. E. Schall, B.Sc. (Lond.). (John Wright and Sons, Ltd., Bristol. 5s.)—To those who intend to take up practical X-ray and allied work, this little book should be extremely helpful. The first part deals with X-rays alone, and the second with general electro-medical procedures. The matter is considered almost entirely from the technical point of view, and upon the physical laws, practical considerations and dosage measurements just that information is provided which is required by beginners and medical students. Interesting comparisons are made between the results obtained with X-rays and with radium. The questions of X-ray sickness and its prevention, and of the various possible accidents and injuries are adequately dealt with. Finally, a series of valuable general, diagnostic and therapeutic tables is provided.

### Materia Medica.

Materia Medica: Pharmacy, Pharmacology and Therapeutics. By Sir William Hale-White, K.B.E., M.D. (J. and A. Churchill. 18th edition. 10s. 6d. net.)—A book popular and successful enough to have reached its eighteenth edition needs none of the reviewer's arts to speed it on its way, and *Materia Medica* is of this type. Beyond remarking that its usefulness is still further increased by several necessary additions and alterations, and drawing attention to the inclusion of a short explanatory account of insulin and its action, there is little to say about this well-known volume, so much used by the medical student, who, after he is qualified, still keeps it as a valuable work of reference. The brief articles—not official—on serums and vaccines, are not the least among its many important and useful contents, while its small bulk and reasonably strong binding render it most convenient for pocket use and frequent handling.

### For the General Practitioner.

The Clinical Examination of the Nervous System. By G. H. Monrad-Krohn, M.D., M.R.C.P. (Lewis. 6s. net.)—This is an excellent and useful little volume. The necessary information as to the clinical examination of the nervous system is given in a concise and clear way. It is essentially a practical book, and it should appeal to the general practitioner and to the student, both of whom are apt to be deterred by the bulk and the diffuseness of a good many volumes dealing with special branches of medicine. It gains in value also from the fact that Dr. Monrad-Krohn, having had experience of mental disorders as well as of neurology in the usual acceptation of the term, is able to incorporate a brief but useful outline of the examination of the mental condition of the patient. There are sections dealing with the examinations of the cerebro-spinal fluid, the Binet-Simon tests, vestibular tests, etc. It may be pointed out that this is not a translation; and we may, therefore, the more congratulate Dr. Monrad-Krohn on his lucid exposition.

### Sonnets and Self-Restraint.

Towards Life, Happy, Healthy, Efficient. By A. Rabagliati, M.A., M.D., F.R.C.S. (Lond.), Humphries and Co. 10s. 6d. net.)—The author of this somewhat remarkable book has set himself the task of delivering what he considers to be a message for the times in the shape of an exposition of the main principles of science, philosophy and religion, in so far as they constitute the threefold manifestation of the unity of Nature. Impressed by the fact that the present tendency of human nature is "to make life easy whether right or wrong," he unfolds the doctrine of self-restraint as being the more excellent way, and through the media of sonnet and prose he shows that the prevention of disease and the attainment of a happy and efficient existence are largely in our own hands. In mal-assimilation of food and in the pernicious practice of resorting to hypnotic drugs, he sees common causes of ill-health, whereas if humanity can be persuaded to live in obedience to the laws of life, and not in defiance of them, there is hope for the future of the race. This is a book to read and ponder over in quiet moments.

### Diseases of the Breast.

Diseases of the Breast. By Willmott H. Evans, M.D., B.S., B.Sc., F.R.C.S. Illustrated. (University of London Press, Ltd. 27s. 6d.)—Dr. Willmott Evans has here endeavoured to describe the modern views in regard to diseases of the breast, and has given his own personal experience and views on many problems which remain unsolved. Modern literature has been freely drawn on, but no new facts are revealed. The subject is treated systematically. The first two chapters deal with the anatomy and physiology and methods of examination; the chapters which follow deal with anomalies of development, Paget's disease of the nipple and chronic infections, while the last two-thirds of the book treat of the innocent and malignant neoplasms. The volume is beautifully and copiously illustrated, many of the illustrations are in colour, and the microscopic pathology is also adequately dealt with. The author is a believer in the parasitic theory of cancer and is inclined to the view that chronic mastitis is due to toxæmia. The book is a valuable addition to the literature on the subject and should appeal to both students and practitioners.

### Insulin Treatment.

The Insulin Treatment of Diabetes Mellitus. By P. J. Cammidge, M.D., D.P.H. (E. and S. Livingstone. 6s. net.)—A particular point of interest in this book is the fact that many of the author's earlier "insulin" cases had already been under accurate laboratory observation for more or less lengthy periods. The conclusions reached are therefore based upon sound scientific methods, even though they may not be entirely in agreement with the generally accepted views on diabetes. A comparison is made between treatment by insulin and by fasting, and in that the various possible modifications of the former are all described, the practitioner should find this particular section especially useful. The question of renal glycosuria and its frequency receives due consideration, and it is noteworthy that as a clinical entity it is found to be not very common, the danger of using insulin in such cases being avoidable by the simple expedient of estimating the blood-sugar level. The action of insulin in providing physiological rest for an over-strained system is well worked out, also the reasons why insulin has not proved to be a "cure" for diabetes in the ordinarily accepted sense of the word. The book is an excellent review of the subject.

### Professional Books of Reference.

The Medical Register, 1924 (General Medical Council) —What *Crockford* is to the clergy and *Who's Who* to the world at large, the *Medical Register* is to the doctor and those who are intimately concerned in the public health. The issue for the current year, published well in time, is as meticulously accurate as ever—we have examined it carefully in hope of enjoying the unholy glee of finding an error. We have failed, and the General Medical Council are to be congratulated upon the exactitude of their Editor. The volume is, however, much more than a list of registered medical practitioners. It contains the full text of all the important Acts of Parliament affecting the profession, together with the fearsome "Warning Notice." The Dentists' Register, 1924 (Dental Board of the United Kingdom)—This Register, which is "swelling wisibly," contains the names and addresses of all practitioners registered in United Kingdom, Colonial and Foreign dentists, together with the text of the relative Acts of Parliament, and a loose inserted list of bodies corporate carrying on "the business of dentistry." The volume is uniform with the *Medical Register* and is marked by the same conspicuous care in compilation.—Medical and Dental Students' Register (General Medical Council)—This useful list of the students registered during 1923 enables the budding doctor or dentist to see himself in the preliminary glories of large type, with the particulars of his preliminary examination in Arts, and his Pre-Registration Examination in Elementary Science. Thus we are in the position of General Wade—we can see something of our doctors and dentists "before they were made."—Minutes of the Dental Board of the United Kingdom for 1923 (Dental Board)—Here we have a complete record of the proceedings, disciplinary and otherwise, of the Dental Board and its various Committees, together with its regulations, the possession of which is essential to every dentist.

